# Restoration Scaling Approaches to Addressing Ecological Injury: The Habitat-Based Resource Equivalency Method

**DOI:** 10.1007/s00267-019-01245-9

**Published:** 2020-01-08

**Authors:** Mary Baker, Adam Domanski, Terill Hollweg, Jason Murray, Diana Lane, Kristin Skrabis, Robert Taylor, Tom Moore, Lisa DiPinto

**Affiliations:** 1 National Oceanic and Atmospheric Administration, 7600 Sand Point Way NE, Seattle, WA 98115 USA; 2 ECONorthwest, Seattle, WA USA; 3 Abt Associates, Boulder, CO USA; 4 Abt Associates (Currently: The Nature Conservancy), Boulder, CO USA; 5 US Department of the Interior, Washington, DC USA

**Keywords:** Natural Resource Damage Assessment, Ecological injury, Habitat, Restoration scaling

## Abstract

Natural resource trustee agencies must determine how much, and what type of environmental restoration will compensate for injuries to natural resources that result from releases of hazardous substances or oil spills. To fulfill this need, trustees, and other natural resource damage assessment (NRDA) practitioners have relied on a variety of approaches, including habitat equivalency analysis (HEA) and resource equivalency analysis (REA). The purpose of this paper is to introduce the Habitat-Based Resource Equivalency Method (HaBREM), which integrates REA’s reproducible injury metrics and population modeling with HEA’s comprehensive habitat approach to restoration. HaBREM is intended to evaluate injury and restoration using organisms that use the habitat to represent ecological habitat functions. This paper seeks to expand and refine the use of organism-based metrics (biomass-based REA), providing an opportunity to integrate sublethal injuries to multiple species, as well as the potential to include error rates for injury and restoration parameters. Applied by NRDA practitioners in the appropriate context, this methodology can establish the relationship between benefits of compensatory restoration projects and injuries to plant or animal species within an affected habitat. HaBREM may be most effective where there are appropriate data supporting the linkage between habitat and species gains (particularly regionally specific habitat information), as well as species-specific monitoring data and predictions on the growth, density, productivity (i.e., rate of generation of biomass or individuals), and age distributions of indicator species.

## Introduction

This paper addresses the scaling of compensatory restoration to address ecological injuries in the context of natural resource damage assessment (NRDA)^1^ under the Comprehensive Environmental Response, Compensation and Liability Act of 1980, 42 USC §9601, et seq. (CERCLA) and the Oil Pollution Act of 1990, 33 USC. §2701, et seq. (OPA). Natural resource trustee agencies^2^ are authorized to assess and recover damages^3^ from potentially responsible parties as compensation for injuries to natural resources that result from releases of hazardous substances (CERCLA) and oil spills (OPA).^4^ The purpose of NRDA is to determine and quantify the extent of the injury, destruction or loss; to calculate and recover the damages needed to compensate for the injury, destruction or loss; and to use the recovered damages to restore, replace or acquire the equivalent of the injured natural resources. The focus of any NRDA is the need to determine how much, and what type of, restoration will adequately compensate the public for the injuries to natural resources. Compensatory restoration is any action taken to compensate for interim losses of natural resources and services that occur from the date of the incident until recovery (15 CFR § 990.30). These actions restore, rehabilitate, replace, or acquire the equivalent resources or services that were lost.

All NRDA claims start with an evaluation of potential injury to natural resources. A conceptual site model can help illustrate how releases could move through the environment, which habitats and species might be exposed to contaminants as a result of the releases, and how potential exposures could injure habitats and species of concern and the services^5^ they provide to the public. Determining the amount and type of restoration that would compensate for injuries to natural resources is an important step in completing an NRDA claim. The amount and type of information practitioners compile to complete a damage assessment varies depending on the size and complexity of the release (e.g., multiple contaminants; long duration; diverse habitats exposed), the type and extent of potential injuries, and other factors (e.g., whether the assessment is being conducted in cooperation with responsible parties (USDOI 2019)).

Relationships between environmental variables and ecological services can be complex, and practitioners should consider the many ecological functions of a habitat when identifying key species that play important roles in delivering ecological services. Various ecological metrics can be used to evaluate the health of species and habitat components. For example, biomass (the weight or total quantity of living organisms of one animal or plant species) is commonly measured over a unit area or volume of habitat. The weight or quantity of organisms in an area at a given moment is the standing crop. The total amount of organic material produced by living organisms in a particular area within a set period of time, called the primary (referring to plants) or secondary (referring to animals) productivity, may be measured in units of mass per square meter per year. The total number of individuals or numbers by age or size class (abundance) can also be measured in biomass over a given area. Contaminants may directly kill plants or animals and result in measurable reductions in biomass, productivity, and/or abundance compared with reference sites. Sublethal effects (e.g., reductions in growth or reproductive rates compared to reference sites) can also reduce biomass, productivity, or abundance of a species, but converting reductions in growth or reproduction to species biomass, productivity, or abundance losses requires additional calculations and models that can vary in complexity. Similarly, predicting improvements in biomass, productivity, or abundance after restoration requires calculations of varying complexity. Models available for these additional calculations include simple production/biomass ratios (which indicates the rapidity with which living material can replace itself (RAMP 2014)) or more complex population demographic models used in fishery or wildlife management (Simpfendorfer 2005; Megry 1988).

NRDA practitioners have applied several tools to determine the amount of compensation needed to address ecological injury to natural resources. Habitat Equivalency Analysis (HEA) and Resource Equivalency Analysis (REA) quantify compensation by equating ecological services or species lost due to contamination with those gained through restoration, without directly estimating financial losses or gains. Economic nonmarket valuation tools, in particular, revealed and stated preference methods (including various types of surveys) can explicitly estimate monetary losses associated with injuries to public natural resources (Adamowicz et al. 2001; Champ et al. 2017; Unsworth and Petersen 1995). Regardless of the scaling method used, practitioners must consider many factors in selecting restoration or compensation to address injury to natural resources, including technical feasibility, cost effectiveness, recovery under different alternatives, and consistency with relevant policies and law (43 CFR § 11.82(d)).

HEA has been applied over many years as an effective tool for resolving liability for natural resource injury as part of the NRDA process in the United States (Ando et al. 2004; Roach and Wade 2006; Israel 2019). This method is used to determine the amount of habitat restoration needed to compensate for ecological service losses over time (Unsworth and Bishop 1994; NOAA 2000; Dunford et al. 2004). HEA is designed to capture the flow of services provided by a given habitat. An important underlying feature of HEA calculations is that the services to be gained through habitat restoration will be of comparable type and quality with those services lost in an injured habitat and appropriate to compensate for those lost due to injury (NOAA 2000). HEA calculates habitat service debits and credits compared with baseline habitat conditions and adjusts them to present-day terms through the application of an economic discount rate. Present-day annual values are summed over the duration of the injury and life of the restoration projects to evaluate cumulative losses and gains in a common currency of present-value ecological service units. For calculating habitat service debits (“injury”), baseline conditions are defined as the environmental conditions that would have existed at a site if the release of hazardous substances or discharge of oil had not occurred (often expressed as a percentage of the services provided by a habitat reference site (NOAA 2000)). In calculating habitat service credits (“restoration”), practitioners estimate “uplift” in conditions relative to those prior to restoration actions (baseline conditions as defined for restoration purposes). If environmental conditions were different in the past, or will vary over time in the future, injury or restoration benefits for past or future time periods can be adjusted as information is available.

To calculate HEA service debits and credits, service losses and gains are converted to a percent change from baseline conditions (from 0 to 100%). Baseline habitat conditions are not synonymous with pristine habitat conditions, but rather, reflect conditions that include changes to habitat caused by other events, exclusive of changes attributable to the release of hazardous substances and discharge of oil. NRDA practitioners may determine percent service losses using injury data from the assessment area (e.g., percent mortality of benthic invertebrates exposed to contaminated site sediment), and/or they may translate site-specific exposure data (such as sediment contaminant concentrations) to injuries to macroinvertebrates, fish, and birds using a suite of data from other sites or from available literature (Cacela et al. 2005). Conditions at reference sites may be used to represent baseline conditions if they are similar to injured sites had the release of hazardous substances or discharge of oil not occurred.

Habitat service debits and restoration credits are typically expressed in terms of discounted service acre years (DSAYs) to account for changes over time and potential differences between the injury and restoration implementation (NOAA 2000). A DSAY is the present-value quantum of total services provided by a single acre of habitat over 1 year. Sufficient restoration is determined to be the number of acres of a particular type of restoration that generates the same amount of DSAYs as were lost due to the injury. This restoration can be directly implemented, or converted to a dollar-based damages estimate. HEA model metrics as service indicators can be used as substitutes for one another (NOAA 2000), with the possibility of one for injury and another for restoration whereby an increase in one metric compensates for a decline in another.^6^ Practitioners may decide to use the HEA approach because of its: (1) focus on services provided by an injured habitat, (2) ability to calculate the amount of habitat restoration needed to replace lost services, (3) flexibility for different types of available data to describe services, (4) familiarity among trustee agencies and potentially responsible parties, and (5) ease of implementation.

The basic equation for a change in DSAYs for a specific habitat type, *h*, takes the standard form:

$$DSAYs_h \,=\, \mathop {\sum }\limits_{t \,=\, 0} \frac{{A_h \cdot \left( {ES_{h,t}^B \,-\, ES_{h,t}^I} \right)}}{{\left( {1 \,+\, r} \right)^t}}$$

where *A*_*h*_ is the acres of habitat, *h*, *r* is the discount rate, $ES_{h,t}^B$ represents the baseline ecological services (as a percentage) provided by the habitat at time *t*, and $ES_{h,t}^I$ are the ecological services (as a percentage) provided by the habitat after it has been injured. Proper application of HEA requires the consideration of the inputs and methods detailed in Table 1.

**Table 1:** Overview of HEA considerations (NOAA 2000)

**1**	Habitat selected for assessment is important to the public and injuries to that habitat can be offset through habitat restoration.
**2**	Each portion of injured habitat assigned the same level of service loss is spatially uniform in services provided and in the type of injury. Each portion of restored habitat assigned the same level of service gain is spatially uniform in type of benefits.
**3**	Restoration projects provide benefits of equivalent or known type and value to offset service losses.
**4**	Duration of injury and recovery period of injured habitat is known or can be estimated. The time for the restored habitat to provide maximum benefits to offset injuries is known or can be estimated.
**5**	Injuries and benefits for injured, pre restoration, and post restoration habitat can be estimated as a percent of baseline conditions. Multiple injuries can be converted to a single percent service loss. Multiple restoration benefits can be converted to a single percent service gain.
**6**	Services across time can be made equivalent through the application of an economic discount rate^a^.

^a^Trustee practitioners apply a fixed, 3% discount rate for both HEA and REA. See Julius (1999) for a more in-depth discussion

REA is an equivalency scaling method where the inputs to service production are biological units rather than the spatial (habitat) units of HEA (Desvousges et al. 2018; Zafonte and Hampton 2007). A common biological unit of measure for REA is the number of individual animals killed, but the arithmetic of equivalency analysis can also accommodate sublethal quantities of biomass lost or any other quantum of lost natural resource. An advantage to REA is that the measures used are often transparent and reproducible. In the case of a biomass-based REA, estimates of dead birds can be multiplied by an average mass/bird to transparently calculate the biomass lost. Using these resource metrics (rather than an index of habitat quality) can lead to results that are readily replicated and may provide for greater ease of exposition. Frequently, when individuals are the biological metric, REA employs a stepwise replacement model^7^ for species killed or injured due to releases of hazardous substances or discharges of oil. A common version of the REA method was first used in the North Cape NRDA case (Sperduto et al. 2003).^8^ REA calculations often involve basic population modeling, including elements of the Leslie matrix (Leslie 1945) and associated life tables (Simpfendorfer 2005). This approach can document how individuals are lost by age class over time based on survival rates and longevity, and determines restoration needed to replace what was lost.

REA calculates resource service debits and credits and adjusts them to present-day terms through the application of an economic discount rate. Injury and restoration are expressed in terms of discounted numbers or biomass of vegetation or animals lost or gained (discounted species years-DSYs). For example, losses to seagrass vegetation can be calculated in terms of discounted kilogram years based on destruction of seagrass cover (the injury), which can be compensated for through restoration projects intended to re-establish vegetation over time (restoration). A DSY is the present value quantum of services provided by the species for one year. As with HEA, an important underlying feature of REA is that the species gained through restoration are of comparable type and quality with the injury, permitting one to evaluate cumulative losses and gains in a common currency of present-value resource units. REA also requires that losses and gains are compared with baseline conditions (the number of individuals or biomass that would be present without the release of hazardous substances or discharge of oil, and the number of individuals or biomass that would be generated in the absence of the restoration action). An important distinction between HEA and REA is that restoration alternatives evaluated by REA are not limited to habitat improvement projects. There may be a broader set of approaches to increase abundance or productivity of injured species (e.g., stocking programs, hatcheries). NRDA practitioners may choose to use the REA model because of its: (1) focus on losses to and replacement of individual species, (2) generation of results that are transparent and reproducible based on directly quantifiable metrics, and (3) ease of implementation.

Injury to a specific species, *S*, is calculated as:

$$DSYs_S \,=\, {\sum \limits_{t \,=\, 0}} \frac{{\left( {N_{S,t}^B \,-\, N_{S,t}^I} \right)}}{{\left( {1 \,+\, r} \right)^t}},$$

where $N_{s,t}^B$ and $N_{s,t}^I$ represent the number of individuals in the population at time *t* under baseline and injured conditions, respectively. *S* can be substituted for the specific species type (e.g., Discounted Bird Years for birds, Discounted Mammal Years for mammals). When population-level estimates are unavailable, the numerator can be simplified to account for the direct and indirect mortality as a result of the injury. As in HEA, *r* represents the discount rate. Both HEA and REA can be adjusted to represent any time interval (e.g., months or days) (Hampton and Zafonte 2006). Table 2 provides information on the REA inputs and methods that allow practitioners to reasonably simplify the analysis by focusing on the dead individuals and associated lost discounted species years (measuring the injury directly).

**Table 2:** Overview of REA considerations

**1**	Species selected for assessment are important to the public and losses can be offset through restoration.
**2**	Injuries to indicator species over time can be measured in meaningful biological units (numbers lost, productivity foregone, etc.).
**3**	Proposed restoration projects are expected to increase the biological units of indicator species measured for injury (or equivalent) over some known or projected timeline.
**4**	Baseline abundance, survival rates, age distributions, fecundity, and other important life history variables are known or can be estimated.
**5**	Biological resources across time can be made equivalent through the application of an economic discount rate^a^.

^a^Trustee practitioners apply a fixed, 3% discount rate for both HEA and REA. See Julius (1999) for a more in-depth discussion

When REA uses continuous biological variables (i.e., the same life history on both the debit and credit), the scaling process mathematically reduces to a simple scalar like in HEA. With more data, more complicated population dynamics of the given resource may be introduced. In applying either HEA or REA models, NRDA practitioners can apply linear loss and recovery curves, or other trajectories based on particular natural resources and site-specific issues.

For both HEA and REA, the costs associated with replacing the lost services are equated to the cost of implementing, overseeing, and monitoring specific restoration projects that are expected to generate the compensatory gain in habitat services or numbers (or biomass) of animals or plants. Cost estimates have also been generated by evaluating “typical” projects or restoration approaches. Since their inception, equivalency methods such as HEA and REA have become the most widely used methods to assess ecological injury and to scale restoration for NRDA.

In addition to HEA and REA, there are other methods available to determine the economic values provided by ecosystem services. These economic methods can use data on the public’s decisions or stated-preferences to derive estimates of these ecosystem service values in monetary units (Adamowicz et al. 2001; Champ et al 2017; Unsworth and Petersen 1995). In 2012 and 2013, the National Research Council issued reports encouraging natural resource trustee agencies to adopt a broad ecosystem services approach to addressing the injuries from the *Deepwater Horizon* oil spill (National Research Council (NRC) 2012, 2013). The ecosystem services valuation approach described by the NRC requires a complex integrated modeling framework to assess losses.

In NRDA cases, a variety of factors, including data quantity and quality, assessment costs, and the ability to address injuries through restoration, are all taken into account when determining how to resolve claims to compensate the public for injuries to natural resources. All quantification methods have advantages and challenges. While the stated preference methodologies in general and the comprehensive ecosystem-based approach described by NRC (2013) in particular, are attractive from an analytical perspective, their complexity and expense may make them impractical for many NRDA cases. HEA has proven to be a flexible, cost-effective, and successful tool to reach settlements, especially where there is a clear habitat injury. REA has been beneficial in cases where individual species are of concern and there have been observable mortalities or clear loss of species reproduction. However, for cases where multiple functional attributes of a habitat have been injured and where identifying the habitat attributes necessary to calculate restoration to compensate for multiple injuries in a single habitat may not be straightforward, a modification of existing methods may provide an additional useful tool. Building on trustees’ experience with biomass-based REAs, the Habitat-Based Resource Equivalency Method (HaBREM) is proposed as an augmentation of existing scaling methods to address complex habitat injuries (including sublethal effects) and allow practitioners to quantify appropriate compensatory habitat restoration. HaBREM is designed to be repeatable, predictable, and technically rigorous. Existing scaling methods often rely on best professional judgment to combine multiple metrics, infer declines or gains in ecological services, and project recovery of services over time. Depending on the context of the NRDA, these assumptions may inhibit agreement between trustees and responsible parties. HaBREM uses a structural approach that limits practitioner inference and relies instead on empirical measures of injury and recovery, which may increase the defensibility of trustee claims.

Examples of HaBREM are provided to illustrate how the method can be used to quantify restoration to compensate for ecological injuries to habitat that are indicated by sublethal effects to multiple species that use the same injured habitat (such as salt marsh). One advantage of HaBREM is that by compiling species-specific analyses of injury and restoration benefits in the same regional habitats over time, it should help NRDA practitioners focus future data collection efforts on the species indicators and endpoints most important for scaling habitat restoration to compensate for injury. The paper proceeds as follows: Section 2 describes the structure for HaBREM and how it can be applied to complex injury and restoration scenarios. Section 3 demonstrates HaBREM by applying it to a hypothetical injury example. Section 4 concludes with a discussion and considerations for practitioners.

## Introduction to Habitat-Based Resource Equivalency Method

The HaBREM methodology assesses service flows from a habitat by evaluating multiple ecological metrics, representing measurable changes in abundance (converted to biomass) or biomass of individual species (or specific life stages of the indicator species) as distinct indicators of injury to the habitat. Ecological services produced by a habitat are calculated through the productivity (rate of generation of biomass) of each of the indicator species metrics (Peterson et al. 2003; Strange et al. 2002). Under HaBREM, any decline in a single ecological metric results in a decline in the overall services of the habitat, regardless of the quantity of the other indicators available. Said another way, an increase in any one metric cannot compensate for a decline in another. Without an explicit measure of substitutability between metrics, this model treats the selected metrics as jointly producing ecological services in fixed proportion.^9^ Some or all of the metrics evaluated can have relationships with each other, and the model does not depend on inclusion of the entire suite of species harmed by a release or oil spill. Because the magnitude of the restoration project is determined by one metric, there is no opportunity to increase restoration project size through redundant metrics (i.e., no double-counting). Selection of more or fewer metrics could lead to different results in HaBREM, but not because of any aggregation. Considerations for selecting appropriate indicator variables that relate to ecological services are reviewed in NOAA (2000); Peterson et al. (2003); Strange et al. (2002); Kandziora et al. (2013).

The HaBREM methodology can provide an appropriate representation of injury and benefits where multiple species or habitat functions have been injured (especially via sublethal impacts) and where identifying the habitat attributes (“driving factors”) needed to compensate for injuries to multiple species may not be straightforward. One of the method’s underlying principles is that when habitat is injured, the individual organisms that rely on and interact with that habitat are also injured, whether the injury represents death or sublethal effects (Cacela et al. 2005). HaBREM uses metrics of injury (loss of abundance or biomass) for a suite of indicator species to represent a habitat-wide injury. Table 3 summarizes inputs and assumptions in applying HaBREM.

In applying HaBREM, the following factors should also be considered:

1. Habitat services are the injuries of interest, assessed through a suite of indicator species, and habitat-based restoration projects are available and appropriate to compensate for injuries. If injury to individual species are of primary interest, separate from any habitat impacts, a traditional REA may be more appropriate. In those cases, species-specific restoration alternatives may be available to more cost-effectively meet trustees’ restoration goals to increase abundance or productivity of individual species of concern (e.g., enhancing populations through propagation in hatcheries and reintroduction).
2. Practitioners should attempt to choose abundance or biomass metrics to be representative of the full suite of functions for the selected resource species and habitat injured by contamination. Practitioners may choose to not measure certain functions since some may not be injured or others may not be measurable through feasible or cost-effective approaches. In selecting from among restoration alternatives, cost effectiveness, technical feasibility, and other factors described in 43 CFR § 11.82(d) must be considered.
3. As is the case with all methods, injury quantification will benefit from development and use of robust conceptual models to evaluate the habitats, functions, and species likely to be injured. Selection of indicator species, target life stages, and endpoints should be based on a connection between releases, pathways, and exposure to target species; the roles of indicator species in providing habitat services; their known sensitivity to relevant site contamination (based on literature or past assessments); determination of the cost effectiveness of data collection and analysis; availability of reference sites and historical baseline data; and availability of habitat restoration actions to compensate for losses. This method will be most effective when the same ecologically relevant indicator species, life stage, and metrics can be evaluated at injured sites and have been monitored over a period of years at restoration sites.
4. Each indicator species metric is calculated individually, for both the injury and restoration side of the equation, using an abundance or biomass measure. Quantifying losses and restoration gains requires information on recovery time (and functional form of recovery) and baseline growth, density, age distributions, and productivity of target species (total biomass generated over space and time) (Peterson et al. 2003; Strange et al. 2002). Although not illustrated here, calculating results for individual metrics allows practitioners to calculate error rates around injury and restoration recovery estimates.
5. Sublethal endpoints can be evaluated by converting into lost productivity (e.g., reduced growth or reproductive output). Similar information on how restoration would benefit productivity will be required for the species and endpoint to be useful (Peterson et al. 2003; Strange et al. 2002).
6. Full compensation of the injury to habitat requires the restoration of the limiting biota (the driving factor selected from among the attributes assessed). Thus, in order to fully compensate for injury to a suite of species connected to the injured habitat, it would be sufficient to complete the amount of restoration necessary to compensate for the species requiring the greatest area (accounting for the severity of injury and time horizon of recovery and restoration of each species). If a lesser area of restoration is completed, then the public would be undercompensated for injury to habitat.

### Basic HaBREM Application

We illustrate the HaBREM methodology by applying it to simplified hypothetical scenarios. The basic application of HaBREM is discussed below in more detail for (1) injury to a single habitat type, and (2) injuries to multiple habitat types.

**Fig. 1:**
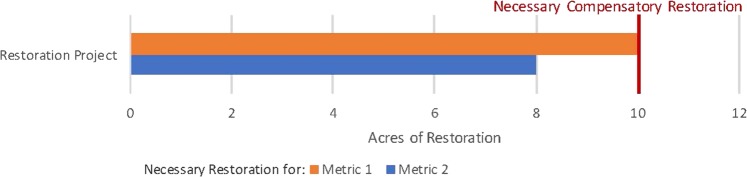
Single habitat injury, multiple metrics, and single restoration alternative. Restoration of salt marsh needed to compensate for all injury to single habitat type (salt marsh). Metric 1 represents fish productivity, metric 2 represents benthic invertebrate productivity

We first demonstrate HaBREM by applying it to a basic scenario with an injury (1) to a single habitat type (coastal salt marsh), (2) measured using multiple indicator species and metrics relative to reference conditions (fish productivity and benthic invertebrate productivity), and (3) that can be compensated with a single type of restoration that restores those multiple injury metrics (planting/enhancing marsh vegetation). In this example, we assume 10 acres of restoration would compensate for the injuries to metric 1 (fish productivity), while 8 acres would compensate for the injuries to metric 2 (benthic invertebrate productivity). Applying HaBREM, the amount of restoration needed to fully compensate for ecological injuries is determined by the indicator species and metric that requires the greatest amount of restoration. Thus, in this scenario, 10 acres of salt marsh vegetation enhancement would compensate for all injuries. Since the 10 acres that addresses injury to metric 1 also restores services represented by metric 2, the restoration needs for each indicator species group are not added together to determine total restoration needed. This concept is visualized in Fig. 1.

**Fig. 2:**
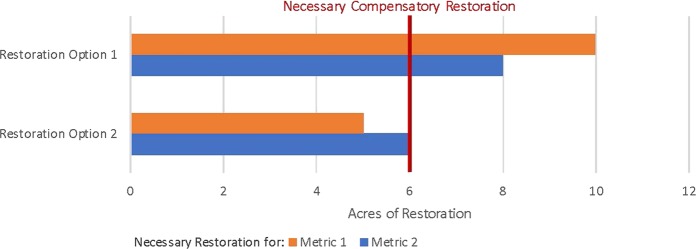
Single habitat injury, multiple metrics, and multiple restoration alternatives. Restoration of salt marsh (replanting or marine debris removal) needed to compensate for all injury to single habitat type (salt marsh). Metric 1 represents fish productivity, metric 2 represents benthic invertebrate productivity

A variation of the forgoing scenario often occurs where injury to one habitat measured using multiple indicator species and metrics can be effectively restored by implementing various types of restoration. The same construct is used, and selection of the individual indicator species and metric that results in the largest area of restoration would compensate for all the other species and the total habitat injury, as displayed in Fig. 2 below. In this example, two restoration project types are capable of compensating for the habitat injury; however, restoration alternative 2 is capable of doing so with fewer acres. A specific example of this case is represented by a contaminated coastal salt marsh where both fish productivity (metric 1) and benthic invertebrate productivity (metric 2) are reduced as compared with reference conditions. In this scenario, losses to the salt marsh represented by both fish and benthic invertebrate productivity can be compensated for with planting to improve a coastal salt marsh (restoration alternative 1) OR through removing debris from a degraded marsh area (restoration alternative 2). The area of coastal marsh planting improvement that generates sufficient fish and benthic invertebrate productivity may be larger than the area of debris removal sufficient to generate the same productivity and compensate for the same injuries.^10^ Thus, in this example, six acres of debris removal (restoration alternative 2) would compensate for all injuries (assuming any differences in total project costs, feasibility, and other factors are acceptable to trustees).

**Fig. 3:**
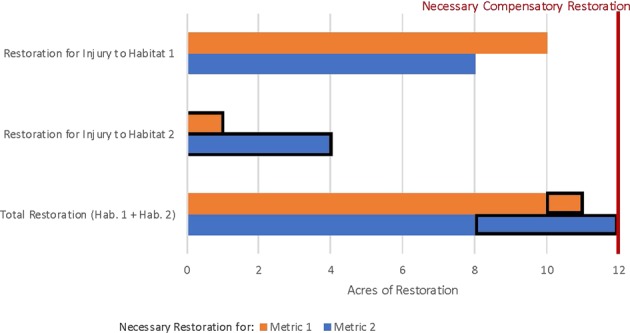
Multiple habitat injuries, multiple metrics, and single restoration alternative. Restoration of salt marsh needed to compensate for all injury to two habitats (salt marsh and mudflat). Metric 1 represents fish productivity, metric 2 represents benthic invertebrate productivity

HaBREM provides additional flexibility for addressing situations with injuries to multiple habitat types and where multiple restoration alternatives may restore for injuries. In this third scenario, there are two distinctly different habitats that are injured, there are two indicator metrics that are common as injury indicators to both habitats, and the injuries to both habitats can be compensated with the same restoration project type. In this situation, the necessary restoration for the two distinct injured habitats are added together for each metric and the amount of restoration that compensates for the metric requiring the largest total restoration area would compensate for all injured species, as displayed in Fig. 3 below. For example, coastal salt marsh (habitat 1) and mudflat (habitat 2) are both injured by a discharge of oil or release of hazardous substances. Fish productivity (metric 1) and benthic invertebrate productivity (metric 2) in the contaminated coastal salt marsh and the contaminated mudflat are reduced compared with appropriate reference sites representing current baseline conditions. For purposes of this example, the ecological services that the indicator species represent are the same in both habitats (e.g., a fish that feeds on mudflat invertebrates can also feed on salt marsh invertebrates). Both the injured fish and benthic invertebrate productivity associated with the contaminated coastal salt marsh and the contaminated mudflat can be compensated for with a coastal salt marsh restoration project (single restoration alternative). Because the fish injured in the coastal salt marsh are in addition to those injured on the mudflat, and the benthic invertebrates injured in the coastal salt marsh are in addition to those injured on the mudflat, the injuries to each species are additive in determining the amount of restoration needed to compensate for losses. The injury metric requiring the largest total area of restoration would compensate for the additive resource injuries from the two separate habitats. Here, the acres of restored coastal salt marsh that compensate for additive injured benthic productivity from the contaminated coastal salt marsh AND from the contaminated mudflats would compensate for fish and benthos injured in the two separate habitats. If practitioners find it acceptable to restore a habitat that is substantially different than the habitat that was injured, the conversion from one habitat type to the other is conducted using relative productivity of the habitats for the specific species considered. However, additional consideration of and justification for how ecological services compare between the two habitats may be beneficial.

**Table 3:** Overview of HaBREM considerations

**1**	Habitat selected for assessment is important to the public and injuries to that habitat can be offset through habitat restoration.
**2**	Multiple indicator species are injured and those injuries over time can be measured in biomass. The metrics evaluated represent important components and functions of injured habitat.
**3**	Proposed restoration projects are expected to increase the biological units (biomass) of indicator species measured for injury (or equivalent) over some known or projected timeline.
**4**	Baseline abundance, survival rates, age distributions, fecundity, and other important life history variables are known or can be estimated.
**5**	Biological resources across time can be made equivalent through the application of an economic discount rate^a^.

^a^Trustee practitioners apply a fixed, 3% discount rate for both HEA and REA. See Julius (1999) for a more in-depth discussion

**Fig. 4:**
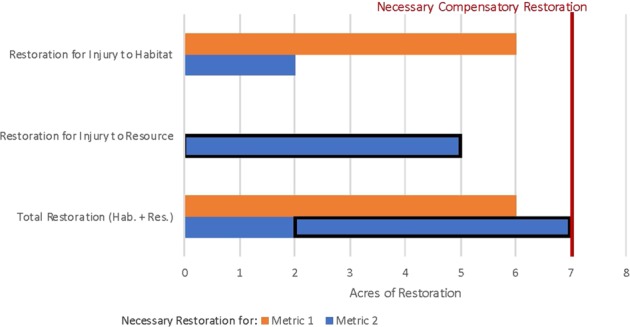
Habitat and resource injuries, multiple metrics, and single restoration alternative. Restoration needed to compensate for injury to habitat and key natural resource species. Metric 1 represents vegetation productivity as an indicator of injury to wetland habitat. Metric 2 represents migratory bird nests as an indicator of injury to wetland habitat. Specific resource injuries (reductions in migratory bird abundance and productivity based on a number of dead oiled birds) were evaluated separately from injury to wetland habitat

Distinct habitat and species-specific injuries that have a single restoration alternative may also occur. In this fourth scenario, a release causes injury to habitat and also distinct injuries to individual important species such as a migratory bird using the habitat. For example, injuries to an oiled habitat are indicated by vegetation productivity (metric 1) and a reduction in numbers of nests for the migratory bird (metric 2), thereby decreasing the bird abundance (or biomass or productivity) compared with baseline conditions. In addition, dead oiled adult birds were collected from the adjacent shoreline. In this example, both distinct injuries can be compensated by the same restoration project. For example, wetland creation may compensate for an oiled wetland with reduced vegetation productivity and a co-occurring reduction in bird nests, as well as a distinctly separate calculation of mortality to adult birds on the shoreline. The need to restore for injuries to the wetland and to compensate for the dead oiled birds are treated separately in the case of the additive species category (total bird biomass losses). Compensation for the additive injuries to the resource and habitat (using the bird metric) is the amount of restoration that compensates for the injury metric indicating the largest area of restoration, as displayed in Fig. 4 below.

## Hypothetical Example Detailed Calculations

To illustrate the calculation of required habitat for restoration using HaBREM, we provide detailed injury and restoration calculations for a hypothetical release. Our hypothetical example proceeds as follows: in 2010, 50 acres of Gulf of Mexico *Spartina* marsh edge were oiled after a nearshore vessel collision. A NRDA was undertaken with numerous field data collection efforts. Average total polycyclic aromatic hydrocarbon (PAH) concentrations were measured in marsh edge soil and used to design laboratory toxicity tests using sediments “spiked” with appropriate concentrations of PAH (in addition to observing injury metrics in the field). Based on the conceptual site model indicating pathways and likely exposure, available data, and trustees’ restoration goals, five indicator species that contribute to marsh productivity were selected and evaluated using different metrics, specifically vegetation (*Spartina* above and belowground biomass), white and brown shrimp (productivity), gulf killifish productivity, and productivity of a representative amphipod species (referred to generically as “amphipods”). All of the species are expected to naturally recover over time. As with many NRDAs, this hypothetical example illustrates practical limitations to data collection. For example, vegetation belowground production measurements were only collected in year 0 and year 7, white and brown shrimp production were measured in year 0 and year 1, and gulf killifish and amphipod production were measured in years 0–3. For years without data, values were interpolated between the closest data points. In addition, years to full recovery are based on data indicating the time when PAH concentrations will likely decline to levels found at reference sites or to concentrations below those significantly toxic in laboratory studies. Once species begin to recover based on reductions in exposure, the time for injured species to return to baseline includes time to full maturity and baseline size distributions for long-lived species (to allow the habitat to develop size distributions present before the spill). Current baseline conditions were determined using an unoiled reference site. Information from the literature and experience from past spills was used to estimate time for different species to reach maturity and reference population size distributions. The injury inputs are summarized in Table 4 below.

**Table 4:** Hypothetical injury^a^

Years since injury	Annual productivity					
	Aboveground vegetation	Belowground vegetation	White shrimp	Brown shrimp	Gulf killifish	Amphipods
	(g dw/m^2^ per year)^b^	(g dw/m^2^ per year)^b^	(g ww/m^2^ per year)^b^	(g ww/m^2^ per year)^b^	(g ww/m^2^ per year)^b^	(g ww/m^2^ per year)^b^
Baseline	2560	1384	38	64	63	39
0 year	1238	878	2	20	1	2
1 year	1805		2	2	1	3
2 years	1432		**38**^**c**^	**64**^**c**^	1	2
3 years	2082				20	11
4 years					**63**^**c**^	**39**^**c**^
7 years		1086				
8 years	**2560**^**c**^	**1384**^**c**^				

^a^While the example we provide is hypothetical, inputs for this table were developed by reviewing previous studies conducted at oiled marsh sites in the Gulf of Mexico and on the East Coast of the United States, including: Michel et al. (2009); Judy (2013); Hester et al. (2015, 2016); and Powers and Scyphers (2016). Although this example is based on realistic conditions found at injured sites in past oil spills, it is intended for purposes of illustration only and should not be used as a basis for potential future claims

^b^G dw/m^2^ refers to grams of dry weight of the target species per square meter; G ww/m^2^ refers to grams of wet weight of the target species per square meter

^c^Values in bold represent expected return to reference conditions in a timeframe determined from other cases

**Fig. 5:**
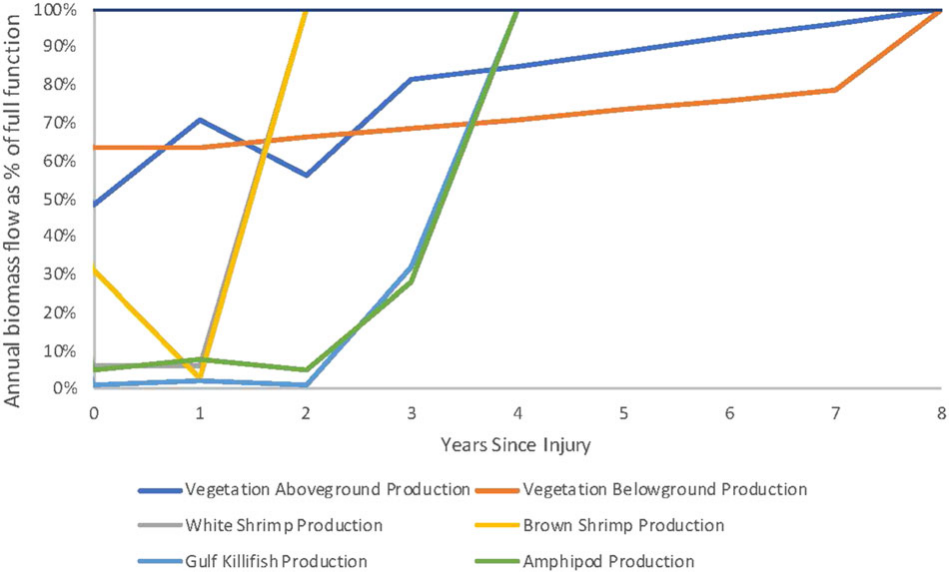
Hypothetical example: Productivity of 50 acres of injured habitat over time

The total productivity of the injured 50-acre habitat over time (as a percentage and relative to baseline and not discounted) is displayed in Fig. 5 below. To apply the HaBREM methodology, measured productivity values were used; efforts have not been made to “smooth” the curves to create an idealized recovery example.

To restore for the injured species, the NRDA practitioners in this example determined that marsh habitat creation is the preferred restoration alternative for initial quantification. As noted above in Fig. 2, there may be other restoration project types that could compensate for the injuries, which could be evaluated through repeated analysis. The created marsh eventually produces the same resources as the injured marsh had produced prior to the oil spill. However, some of the species produced by the created marsh quickly achieve full productivity when compared with a reference marsh, while other species experience a delay in achieving full productivity. Restoration actions will be delayed until 5 years after the spill while the trustees complete the assessment, negotiate with potentially responsible parties, and conduct necessary restoration planning and permitting. Based on information from other restoration efforts, once constructed, the created marsh will take up to 18 years to provide full function for all species indicators and is expected to persist and convey creditable benefits for a total of 20 years after restoration implementation due to local sea level rise and subsidence. Conditions at a reference marsh are intended to represent fully functional restored marsh conditions. Uplift is calculated from the starting conditions at the restoration site before the project is implemented.

**Fig. 6:**
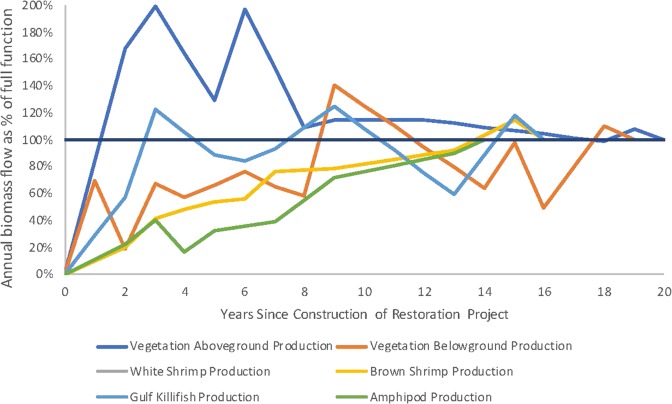
Hypothetical example: Productivity of restored habitat over time. Lines depicting white shrimp production and brown shrimp production partially overlap due to the use of the same hypothetical recovery curve

The practitioners in our hypothetical example conducted a thorough literature review to determine recovery rates and the expected productivity achieved for each of the metrics for their restoration crediting and planning effort. The literature indicated that for created marshes in this region, it is typical to see elevated aboveground vegetation productivity in the initial years of restoration, and then a decline to reference values. As with the injury data, the practitioners used all appropriate reported values from the literature and did not “smooth” the curves to create an idealized recovery trajectory. For the interim years without data, practitioners interpolated between the closest data points. Once each species reached full productivity, that reference value was used for the remaining years of the restoration project. The modeled productivity data for metrics at the restored site over time (uplift only) is displayed in Table 5. The calculated productivity of the restored habitat over time (as a percentage of uplift only and not discounted) is displayed in Fig. 6 below.

**Table 5:** Hypothetical restoration^a^

Years restored	Annual productivity					
	Aboveground vegetation	Belowground vegetation	White shrimp	Brown shrimp	Gulf killifish	Amphipods
	(g dw/m^2^ per year)^b^	(g dw/m^2^ per year)^b^	(g ww/m^2^ per year)^b^	(g ww/m^2^ per year)^b^	(g ww/m^2^ per year)^b^	(g ww/m^2^ per year)^b^
0.5 year		588				
1 year		964				
2 years	2863	259	8	13	36	8
3 years	3399	923	16	27	77	16
4 years		788				7
5 years	2197		20	35	56	13
6 years	3357	1047	21	36	53	
7 years		904	29	49	59	15
8 years	1860	803				
9 years	1959	1942	30	50	78	28
13 years			35	59	37	35
14 years		887				**39**^**c**^
15 years		1355	43	73	74	
16 years		685	**38**^**c**^	**64**^**c**^	**63**^**c**^	
18 years	1681	1528				
19 years	1845	**1384**^**c**^				
20 years	**1709**^**c**^					

^a^Similar to the injury data, the restoration inputs are based on previous studies conducted at marsh restoration sites along the Gulf of Mexico and East Coast, including: Minello and Zimmerman (1992); Minello et al. (1994); Sacco et al. (1994); Minello and Webb (1997); Brusati et al. (2001); La Peyre et al. (2009); Edwards and Mills (2005); Shafer and Streever (2000); and Hollweg et al. (2019). These values are intended for example purposes only and should not be used as a basis for potential future claims

^b^G dw/m^2^ refers to grams of dry weight of the target species per square meter; G ww/m^2^ refers to grams of wet weight of the target species per square meter

^c^Values in bold represent expected return to reference conditions in a timeframe determined from other cases

By calculating each metric independently, HaBREM can determine the quantity of restoration that compensates the public for the overall injury to the habitat. As shown in Table 6, each metric is measured and scaled individually, providing a separate determination of compensatory acres of wetland for each metric. To get from Table 5 to 6, one applies the REA formula for each biota, *S*, (columns in Table 5 and rows in Table 6) to the injury for that biota (each column of Table 4). Table 4 gives the injury portion of the REA:

$$DSYs_S \,=\, {\sum \limits_{t \,=\, 0}} \frac{{\left( {N_{S,t}^B \,-\, N_{S,t}^I} \right)}}{{\left( {1 \,+\, r} \right)^t}}$$

**Table 6:** Metric-specific restoration compensation

Metric	Compensatory acres of marsh
Vegetation aboveground production	7.8
Vegetation belowground production	10.8
White shrimp production	10.0
Brown shrimp production	8.9
Gulf killifish production	14.9
Amphipod production	21.1

Table 5 provides the restoration gains *per acre*:

$$DSYs_S \,=\, {\sum \limits_{t \,=\, 0}} \frac{{\left( {N_{S,t}^{Gained/acre}} \right)}}{{\left( {1 \,+\, r} \right)^t}}$$

Dividing each injury by each gain per acre for each biota, *S*, we get required acres listed in Table 6.

$$Required \, acres \,=\, \frac{{LostDSYs_S}}{{GainedDSYs_S/Acre}} \,=\, \frac{{{\sum \nolimits_{t \,=\, 0}} \frac{{\left( {N_{S,t}^B \,-\, N_{S,t}^I} \right)}}{{\left( {1 \,+\, r} \right)^t}}}}{{{\sum \nolimits_{t \,=\, 0}} \frac{{\left( {N_{S,t}^{Gained/acre}} \right)}}{{\left( {1 \,+\, r} \right)^t}}}}$$

**Fig. 7:**
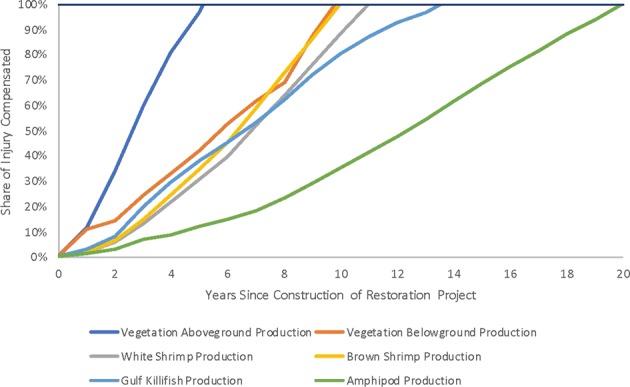
Hypothetical example: Time to compensate for each injury metric using a 21.1 acre Marsh creation project

Using HaBREM, 21.1 acres of created marsh would compensate for all the injured species and losses to the habitat. Upon construction of a 21.1 acre marsh with a 20-year lifespan, some species reach a level of productivity necessary to compensate for injury sooner than others. For a 21.1 acre restoration project, the time at which each metric-specific injury is fully compensated is identified in Fig. 7 below. The figure scales the needed productivity for each species on a scale of 0–100% of the total needed to offset injury for that category.

It is important to note that the compensatory acres in each row of Table 6 should not be interpreted as independent identical signals of a single habitat injury. Rather, each biota represents its own dimension of loss. Each of these losses can be restored by a single habitat type but each biota has its own habitat quantity requirement. HaBREM highlights that full compensation of the injury requires the restoration of the limiting biota, in our example, the amphipod. Our example abstracts from uncertainty by using literature values; an applied case with injury and restoration studies may require explicit consideration of uncertainty in the final selection of the required restoration quantity. But consideration of such uncertainty would maintain the focus on the upper end of the joint distribution of the acreage estimates unless they are all nearly identical. Referring to Table 6, the common tendency in HEA to average across all resources (i.e., restoring 12.25 acres) can be objectively demonstrated to undercompensate for Gulf killifish and amphipods using HaBREM.

**Fig. 8:**
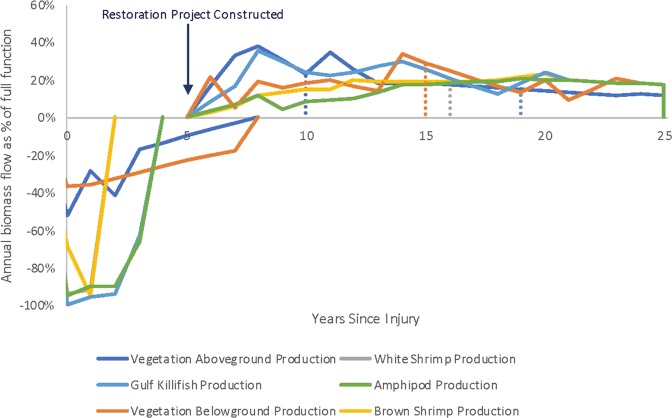
Hypothetical example: Injury to 50 Marsh acres offset by compensatory restoration from 21.1 acres of Marsh creation. Dashed lines indicates time at which compensation for each resource would be sufficient, however the marsh continues to produce all species over its total lifespan

Multiple factors can determine which resource metric will control the amount of restoration needed to compensate for injuries to the habitat. The severity of the injury (total biomass lost and discounted over time) and duration of the injury for each resource metric are indicated by the area above the curves (below the *x*-axis) on the left portion of Fig. 8. The magnitude of the benefit (total biomass gained and discounted over time) of the restored habitat and time to reach full function for each metric are indicated by the area below the curves (above the *x*-axis) on the right side of Fig. 8. The restoration curves leave the *x*-axis at the time the restoration project is initiated (which is before some of the injuries are fully recovered, e.g., vegetation above and belowground biomass). The severity and duration of the injury and the magnitude and time to reach benefits interact in ways that may not be immediately intuitive. For example, practitioners may have originally assumed that brown and white shrimp would behave similarly and would be surrogates for each other. But because the injury to white shrimp was more severe in the first year (the two species settle on the marsh in different seasons), and because an acre of restored marsh produces more brown shrimp than white shrimp, the amount of restoration needed to compensate for injury is different for the two species. In addition, because of varying habitat requirements, life spans, and age class demographics, the species that is most sensitive to contamination may not be the species that needs the greatest area or amount of time to generate compensating total discounted biomass through habitat restoration.

**Fig. 9:**
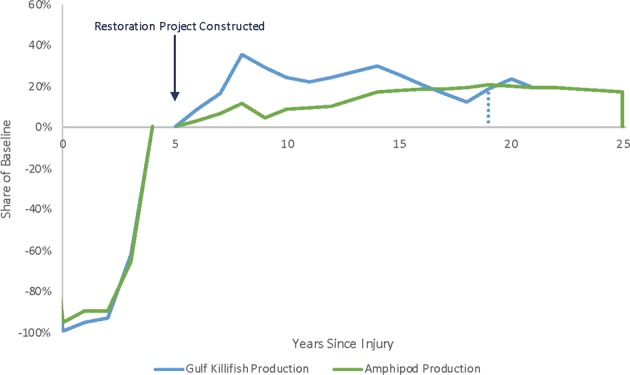
Hypothetical example: Injury to 50 Marsh acres offset by compensatory restoration from 21.1 acres of Marsh creation—Killifish and Amphipods. Dashed line indicates time at which compensation for lost killifish production would be sufficient; however, the marsh continues to produce killifish over its total lifespan. The restoration project is assumed to begin 5 years after the oil spill and remain in existence for a total of 20 years

Closer examination of the injury and restoration paths for killifish and amphipods in Fig. 9 shows that although they have similar severity and duration of injuries, the restoration project generates a lower productivity value for amphipods. Therefore, in this example, amphipods would be the driver to calculate compensatory restoration for all injured species and the habitat as a whole.

Generalized equations to perform calculations are provided in Supplementary Material Appendix A.

## Discussion

The purpose of this paper is to introduce HaBREM as an NRDA method intended to evaluate metrics for organisms that utilize a habitat to represent total habitat function and services over time. NRDA claim development for habitat services consistently face the challenge of limited resources, time, and data. As described above, HaBREM seeks to expand and refine the use of organism-based metrics (as in biomass-based REA) with HEA scaling concepts, providing an opportunity to use data relating to sublethal injuries to multiple species and the ability to measure error rates to develop NRDA claims^11^. When an injury to a habitat occurs, practitioners may be able to use the HaBREM approach to identify the target species, life stages, and metrics most likely to determine the amount and type of restoration needed, and focus financial and staff resources on filling most important data gaps.

Methodologically, HaBREM expands the NRDA claim development toolbox by evaluating ecological services produced by a habitat by focusing on the abundance and productivity (measured as biomass) of biota using the habitat (Peterson et al. 2003; Strange et al. 2002). The amount of restoration needed can be determined by the biological resource that requires the greatest area of restored habitat (if all other restoration selection criteria are acceptable and the varying timeframes of restoration have been taken into account). It is possible that more or fewer metrics could lead to different results in HaBREM, but not because of any double-counting through the selection of redundant metrics. While the selected metrics may have relationships with each other, they are not perfect substitutes. The HaBREM methodology conducts independent evaluation of each metric and seeks to understand its relative value in driving the need for compensatory restoration. A strict adherence to the definition of relative value, which is dependent on the relative scarcity of services and availability of substitutes, means that a decline in one input cannot be offset by an increase in another. Using this definition, restoration for the metric requiring the largest area will also generate a sufficient quantity for the other metrics evaluated. This is consistent with trustees’ focus on restoration projects that can cost-effectively restore for multiple injuries.

HEA, REA, and HaBREM approaches apply the same fundamental framework to balance losses and gains and convert restoration needs to present values using discounting. Contrasts between model attributes are summarized in Table 7.

**Table 7:** Model attributes

	HEA	REA	HaBREM
Purpose	Address habitat injury	Address species injury	Address habitat injury
Injury output units	Discounted Service Acre Years (DSAYs)	Discounted numbers, life stage equivalents, or biomass	Discounted production numbers, life stage equivalents, or health (sublethal effects on organism)—all measured in biomass
Restoration output units	DSAYs (can be converted to acres or other spatial units)	Discounted numbers, life stage equivalents, or biomass (can be converted to acres or other spatial units)	Acres or other spatial units
Basis of injury	% of baseline services (often based on reference site)	Injury output units (e.g., number of individuals) relative to reference site or historical values	Injury output units (e.g., biomass) relative to reference site
Basis of restoration	% uplift relative to baseline services	Uplift in numbers and/or life history-related conditions (restoration output units)	Uplift in biomass-based conditions (restoration output units)
Unique Features	Converts comprehensive habitat features (perhaps representing multiple services) to single percent service loss/gain by evaluating components within the habitat	Applies a reproducible/ measurable individual species focus	Addresses multiple measurable habitat injuries and calculates benefits of habitat restoration needed to compensate for them

In HEA, ecological services are generated by a production function of discrete ecological components, represented by biological metrics. The total production of ecological services is a function of the quantity and marginal productivity of all of the components and metrics (where each could become inputs to an integrated model of total ecological service) (Peterson et al. 2003; Strange et al. 2002). In HEA, ecological services are scaled to 100% of baseline (for injury, these are the conditions that would exist without the release or discharge; for restoration, these represent conditions without the restoration effort).

In REA, ecological services are produced by a single input, the existence of the species in question (i.e., number of animals, or quantity of biomass in life-stage equivalents). Loss of that animal or biomass results in the loss of ecological services. Where multiple species have been killed, practitioners may group species by guilds or use surrogate species to help manage the number of REA runs and associated assessment costs.

As discussed above, HaBREM is intended to evaluate services provided by the habitat over time using metrics representing some aspect of ecological function using individual species or species groups as indicators of total habitat function. It avoids starting with the question of overall percent total services lost and does not require restoration benefits to be framed in terms of percent services.

A conceptual site model should indicate the environmental pathways by which a contaminant release reaches natural resources of concern, the media (e.g., through sediment or surface water) and mechanisms (e.g., dietary uptake or dermal adsorption) by which habitats and specific species are exposed, and injury endpoints applicable to the contaminants, potentially injured species and their sensitive life stages. Such a model will assist practitioners in determining whether a quantification approach that assesses injury to habitats (such as HEA or HaBREM) or a method quantifying injury to specific species (REA) would be more appropriate. A conceptual site model will also suggest potential species and metrics for quantifying injury and whether and how they can be converted to percent services or would serve as quantifiable indicators of lost/gained productivity. If practitioners elect to evaluate injuries to habitat using HaBREM, selection of more than one species and injury metric representing different biological components of the habitat (ideally in units of productivity) will allow practitioners to document how the habitat has been affected and how restoration will compensate for injuries. Identification of habitat restoration alternatives should occur early in the injury assessment process so that benefits of restoration can be quantified using the same species, metrics, and units as injuries.

Once appropriate restoration scaling methods, assessment metrics, and restoration project alternatives are identified, a range of options for compiling appropriate data to apply the HaBREM methodology exist. Directly measuring lost productivity in habitats exposed to a release or discharge is rarely attempted in our experience (e.g., French McCay and Rowe 2003; Jones and Donlan 2008). Field observations (e.g., comparing density of snails in an oiled marsh to those in reference sites) or laboratory toxicity test results (e.g., measurement of reduction in growth of fish held over contaminated sediment) can be converted to decreases in productivity using data from reference areas, life history information from similar sites, and well-established population modeling techniques (Strange et al. 2002; Megry 1988; Simpfendorfer 2005). In the example used in this paper, annual marsh vegetation productivity declines were calculated using measurements of end-of-season standing stock biomass at the injured site and turnover rates from other marshes in the region. The turnover rate is the inverse of the amount of time required for growth and photosynthesis to replace the loss or transport of vegetation out of the habitat (Dettman 2013). In determining the recovery time for injured sites, practitioners consider and apply data from other releases, since the data will not be available at the injured site for many years. The functional form used to simulate recovery has typically been linear, but does not need to be. Applying a range of values in models and injury calculations could allow for sensitivity analysis and calculation of error rates for productivity losses.

Quantifying benefits of proposed restoration projects requires information from prior projects (ideally with similar environmental conditions and in the same region). Restoration monitoring data are often related to the development of structural components such as plant cover or reef height, while biomass or productivity measurements may be scarce datasets. The duration of restoration monitoring also varies widely by project type and region but is often relatively short. Improving values for maximum productivity and time to full function will require additional targeted monitoring efforts over time. Meanwhile, practitioners may assume that restoration projects will ultimately generate “maximum” species-specific productivity values found at *reference* sites, or that the highest productivity values measured over time at other *restoration* sites may represent “full function”. As additional regionally specific monitoring data become available indicating how habitat functions and specific species develop with each restoration action, the evaluation of factors influencing benefits will improve.

Determining injury and quantifying restoration needs requires that practitioners make important decisions regarding what species and metrics to assess and which methods to use to evaluate losses and gains. While practitioners must make further choices regarding what data to include in converting site-specific information to quantifiable losses and gains, clearly documenting decision rules and data sources will increase transparency and reproducibility of scaling methods. For example, practitioners may adopt a specific set of rules for how to treat contamination data that are below detection limit values, particularly for historic data where detection limits may be higher than for recently analyzed data. As another example, practitioners may parameterize a van Bertalanffy growth curve for a specific species using laboratory toxicity data to enable conversion from toxicity test results to biomass reductions.

In summary, the potential benefits of applying the HaBREM methodology include:

1. Focusing on the productivity (measured in biomass) of multiple injured species in a given habitat, including sublethal effects.
2. The ability to quantify estimation error rates for productivity losses and gains, regardless of whether they are calculated using field data from the affected site or data from other sites. The resulting error rates in support of a sensitivity analysis can help ensure the reliability of results.
3. Identification of the specific driving factors for calculating restoration needs which can help support the reliability and technical rigor of restoration scaling.
4. Improved understanding of relationships between productivity of injured and restored species, which will allow future assessments to focus on data collection for variables that most influence results. The data generated by applying HaBREM could be used in HEA development to help expedite future analyses, just as the outputs of REA can be used to develop inputs that are more reliable for HEA.

## Supplementary information

Supplementary Information

## Footnotes

1 Also known as natural resource damage assessment and restoration (NRDAR).

2 Natural resource trustee agencies are designated and their responsibilities described in the National Contingency Plan (NCP), 40 CFR §§300.600-300.615.

3 The term “damages” here is used in the legal sense of monetary compensation for harm, whether that monetary compensation is provided through financial instruments or in-kind restoration.

4 Implementing regulations for NRDA have been adopted for CERCLA by the U.S. Department of the Interior (43 CFR Part 11) and for OPA by the National Oceanic and Atmospheric Administration of the U.S. Department of Commerce (15 CFR Part 990). Compliance with either set of regulations is optional, but NRDA conducted in accordance with the applicable regulations is entitled to a rebuttable presumption in the event of litigation (42 USC § 9607(f)(2)(c); 33 USC § 2706(e)(2)).

5 “Services” in the context of NRDA are defined as “the physical and biological functions performed by the resource including the human uses of those functions” (43 CFR § 11.14(nn)). Services result from “the physical, chemical, or biological quality of the resource” (43 CFR § 11.14(nn)).

6 This mirrors the economic definition of a Cobb-Douglas production function (and other production functions that allow substitution between inputs), where the amount of output produced is a function of multiple inputs, each with a respective output elasticity. For more information, see Mas-Colell et al. (1995).

7 Term coined by Hampton and Zafonte in the *Luckenbach Final DARP*, Appendix C (Natural Resource Trustees 2006), which describes how lost individual years are calculated by the age classes over time in a stepwise fashion. The stepwise concept reflects the Leslie Matrix used by biologists/ecologists. Similar terms are seen in the economics and political science literature to describe various trajectories over time.

8 World Prodigy and BT Nautilus oil spills are early examples of trustees assessing the relationship between bird losses and nest protection as precursors to today’s REA.

9 An explicit function of the relative supply and demand for the services provided by individual species or metrics provides the measure of substitutability. This joint production mirrors the economic definition of a Leontief production function where output is produced in fixed proportion to a set of factor inputs (Mas-Colell et al. 1995).

10 For purposes of the example shown in Fig. 2, it is presumed that both types of restoration project fully address the ecological services lost as a result of the incident in question and that the debris removal project is capable of generating higher productivity per acre.

11 This paper does not demonstrate the calculation of error rates since the example uses summarized values pulled from the literature. The suggested HaBREM application would rely on site-specific data and studies for which errors of each parameter estimate would be available through traditional direct estimation or any variety of resampling methods for more complicated models. The key difference for HaBREM is that any biota specific metrics will be amenable to estimation of uncertainty whereas traditional service loss/gain calculations in HEA are not typically the result of a formula or measurements that would allow for uncertainty analysis.
