# Supplementary material for: Restoration Scaling Approaches to Addressing Ecological Injury: The Habitat-Based Resource Equivalency Method
Source: Environ Manage. 2020 Jan 8;65(2):161–77. doi: 10.1007/s00267-019-01245-9 (PMC7007898; doi:10.1007/s00267-019-01245-9)
Supplement: Supplementary file 1 — Supplementary Information [file 267_2019_1245_MOESM1_ESM.docx]

Supplemental Material **Appendix A: HaBREM Computation**

1. Goal: Calculate restoration necessary to offset injury. For a single habitat, *h*, whose ecological services are measured via a single metric, *m*, solve for $A_{h,r}$ such that:

$$\sum_{t=0}^{T} \frac{A_{h,i}\cdot\left( {\Delta ES}_{m,i,t}^{I} \right)}{{(1+d)}^{t}}=\sum_{t=0}^{T} \frac{A_{h,r}\cdot\left( {\Delta ES}_{m,r,t}^{R} \right)}{{(1+d)}^{t}}$$

Where

- $A$ – Acres of habitat.
- $ES$ – Ecological Services, indexed by:
  - $I$ – Injury, and
  - $R$ – Restored.
- $d$ – Discount rate.
- $h$ – Habitat type.
- $m$ – Habitat metric in set of available metrics, $M$.
- $i$ – Injury.
- $r$ – Restoration.
- $t$ – Time.

1. Rearranging equation 1 results in:

$$A_{h,r}=\sum_{t=0}^{T} \frac{\left( {\Delta ES}_{m,r,t}^{R} \right)}{{(1+d)}^{t}}\cdot\left( A_{h,i}\cdot\sum_{t=0}^{T} \frac{\left( {\Delta ES}_{m,i,t}^{I} \right)}{{(1+d)}^{t}} \right)^{-1}$$

2. Ecological Services have unique and equivalent values by metric, such that:

$${V\left( {\Delta ES}_{m,,t}^{R} \right)}_{t}={V\left( {\Delta ES}_{m,r,t}^{I} \right)}_{t}$$

3. Using the equivalent value assumption in #3, the change in Ecological Services is directly measured by a change in some type of biological measure (i.e. biomass productivity), *b*, such that:

$${\Delta ES}_{m,i,t}^{I}=b_{m,i,t}^{I_{0}}-b_{m,i,t}^{I_{1}}$$

$${\Delta ES}_{m,r,t}^{R}=b_{m,r,t}^{R_{1}}-b_{m,r,t}^{R_{0}}$$

Where

- $b^{I_{0}}$ – biomass productivity but-for the injury (baseline),
- $b^{I_{1}}$ – biomass productivity at injured site,
- $b^{R_{0}}$ – biomass productivity but-for the restoration action (unrestored), and
- $b^{R_{1}}$ – biomass productivity at restored site.

1. When multiple metrics are available, the acres of habitat restoration necessary becomes metric dependent, such that

$$A_{h,r,m}=\sum_{t=0}^{T} \frac{\left( b_{m,r,t}^{R_{1}}-b_{m,r,t}^{R_{0}} \right)}{{(1+d)}^{t}}\cdot\left( A_{h,i,m}\cdot\sum_{t=0}^{T} \frac{\left( b_{m,i,t}^{I_{0}}-b_{m,i,t}^{I_{1}} \right)}{\left( 1+d \right)^{t}} \right)^{-1}$$

2. To identify the metric that drives the restoration requirement:

$$\underset{m}{\mathrm{argmax}} {A(m)}_{h,r}=\left\{ \sum_{t=0}^{T} \frac{\left( b_{m,r,t}^{R_{1}}-b_{m,r,t}^{R_{0}} \right)}{{(1+d)}^{t}}\cdot\left( A_{h,i,m}\cdot\sum_{t=0}^{T} \frac{\left( b_{m,i,t}^{I_{0}}-b_{m,i,t}^{I_{1}} \right)}{\left( 1+d \right)^{t}} \right)^{-1}|\forall m\in M \right\}$$

3. The restoration requirement is defined as:

$$\max_{m} {A(m)}_{h,r}=\left\{ \sum_{t=0}^{T} \frac{\left( b_{m,r,t}^{R_{1}}-b_{m,r,t}^{R_{0}} \right)}{{(1+d)}^{t}}\cdot\left( A_{h,i,m}\cdot\sum_{t=0}^{T} \frac{\left( b_{m,i,t}^{I_{0}}-b_{m,i,t}^{I_{1}} \right)}{\left( 1+d \right)^{t}} \right)^{-1}|\forall m\in M \right\}$$
